# Prandtl and Ohnesorge numbers dependent of ultrasonic horn energy in Newtonian liquid under batch and continuous flow

**DOI:** 10.1016/j.ultsonch.2024.106869

**Published:** 2024-04-03

**Authors:** Jamshid Behin, Hessamodin Shahabazi

**Affiliations:** a Faculty of Petroleum and Chemical Engineering, Razi University, Kermanshah, Iran; b Artificial Intelligence Division, Advanced Chemical Engineering Research Center, Razi University, Kermanshah, Iran

**Keywords:** Ultrasonic waves, Thermal energy conversion, Two-phase homogenous mixture, Causal diagram, Continuous flow, COMSOL Multiphysics^@^

## Abstract

The level of knowledge on the non-thermal contribution of ultrasonic wave’s energy to perform physico-chemical phenomena is one of the bottlenecks for the commercialization purposes. Under constant nominal power of transducer (**P_n_**), the input electrical power (**P_in_**) is less and sensitive to the medium’s physical properties. This study attempts to assess the conversion of acoustic to thermal power experimentally and numerically using COMSOL Multiphysis@ for a 24 kHz horn-type sonicator through a medium without any sono-chemical effect. Single- and homogeneous two-phase Newtonian mixtures of sunflower oil and water (**o/w**) with a relatively wide range of density (914–998 $kg/m^{3}$) and viscosity (0.5–63.5 mPa.s) were irradiated in a lab-scale vessel (1 L) under batch and continuous flow configuration. The direct influence of **P_n_** (80–400 W) and **o/w** ratio (0–1) on temperature rise and subsequent thermo-physical properties of liquid and the indirect influence on **P_in_** and thermal energy conversion (**TEC**) were investigated employing calorimetric method. A new engineering concept including a power factor correlation was proposed and validated for prediction of **P_in_** as a function of liquid space velocity (ϑ), temperature, Prandtl (**Pr**) and Ohnesorge (**Oh**) dimensionless groups. The results showed that under constant temperature and **P_n_**, increasing **Pr** and **Oh** increased **P_in_** with a similar trend for both modes of operation. An increase in temperature directly led to a decrease in **P_in_** with a power factor closed to “-1”. The **P_in_** in continuous flow was higher compared to batch configuration at similar temperature, liquid properties, and **P_n_**. This effect was more significant with increasing ϑ. An increase in ϑ at constant **P_n_** led to a decrease in the inlet/outlet temperature difference in continuous flow and an increase in **P_in_**. Increasing **P_n_** resulted in higher **TEC** for both configurations; however, **TEC** was relatively lower in continuous flow than batch configuration indicating more efficient sonication in continuous flow.

## Highlights

• Energy conversion in a horn type sonicator was investigated under batch and continuous flow.
• Acoustic power was varied depending on the liquid properties at a fixed nominal power of transducer.
• A power relation was proposed for acoustic power prediction as a function of dimensionless groups.
• Temperature, **Pr** and **Oh** numbers are the mains factors affecting acoustic to nominal power ratio.
• Lower thermal energy conversion was obtained in continuous flow compared to batch configuration.

## Graphical abstract

**Figure:**
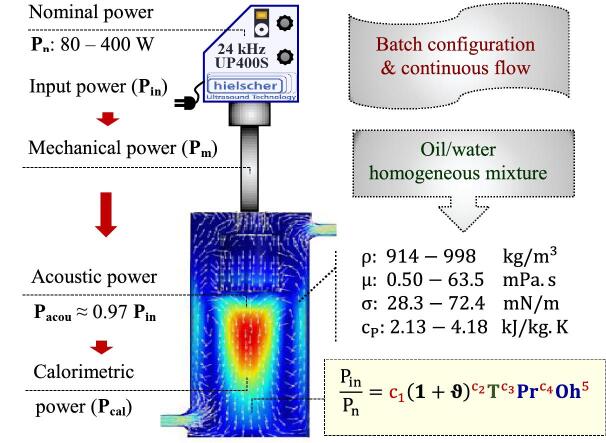

## 1 Introduction

Transducing the electrical pulses (energy) in a piezoelectric crystal generates mechanical vibrations in a liquid medium (acoustic energy) which is known as ultrasonic waves (USW). They can be classified into low (<200 kHz), medium (0.2–2 MHz), and high-frequency (>2 MHz). The propagation of USW from 20 to 800 kHz through a liquid enables the formation, growth, and sudden collapse of microbubbles (∼10 μm in diameter) within a few μs. This phenomenon (acoustic cavitation) creates elevated local pressures (70–100 MPa) and temperatures (>5000 K) known as hot spots. The cumulative effect results in the release of energy into the liquid and is responsible for performing the desired physical (micro-jetting), chemical (highly reactive species), and biological phenomena (tissue damaging) [1]. It can be used for treatment of water [2] and wastewater [3], food processing [4], chemical and nanomaterial synthesis [5], [6], clinical/therapeutic applications [7], fuel refining and oxidative desulfurization [8], polymer welding [9], heat transfer enhancement [10], [11], [12], surface cleaning and sterilization [13], descaling and nondestructive testing. In large-scale sonoreactors with low-frequency range, large-amplitude waves change the physicochemical properties of liquid [14] and disperse acoustic energy more, while in the environment with high-frequency range, sonochemical effects are more significant [15].

**Fig. 1:**
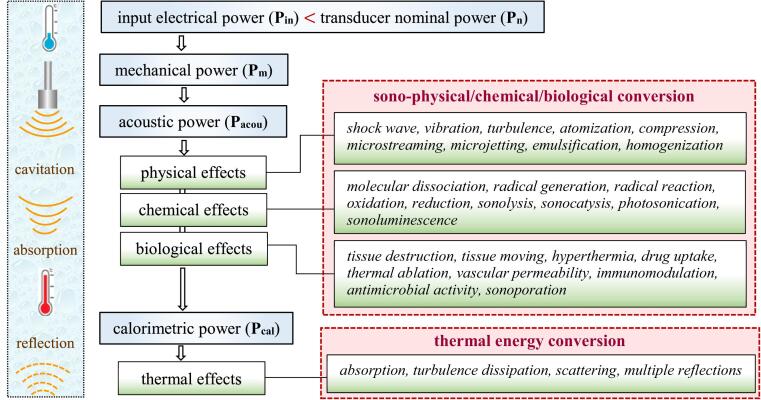
Various effects of USWs and the energy conversion road.

Apart from use of USW energy for preforming the appropriate physico-chemical process, e.g. activation of reactant (Fig. 1), several phenomena, such as molecular absorption, turbulence dissipation, scattering and multiple reflections by the complex process of cavitation result in complete dissipation of the acoustic energy into heat and inappropriate phenomena, e.g. temperature raising, streaming, micro-jetting, shocking, surface erosion, acceleration of by-product formation as well as auto-reduction of delivered acoustic energy [16]. Apart from transducer losses (∼2–4 % input power) of different natures such as electrical and mechanical resistances, the power requirement (acoustic power) for a desired physicochemical conversion is a crucial parameter for practical application at large scale and continuous flow. For this purpose, thermal energy conversion (TEC) is defined as the ratio of calorimetric power ($P_{cal}$) to the acoustic power ($P_{acou}$) or to input electrical power ($P_{in}$)[14].

Reactor geometry and size, US generator frequency (f), pulsation time and nominal power ($P_{n}$), liquid temperature (T) and hydrostatic pressure (**P**) as well as temperature-dependent liquid properties such as vapor pressure ($P_{v}$), compressibility (K), density (ρ), dynamic viscosity (μ), surface tension (σ), specific heat capacity ($c_{P}$), and thermal conductivity (k) are the most important variables affecting $P_{in}$, TEC and sonication efficiency. Batch-mode experiments have demonstrated that the $P_{in}$ and sonication efficiency reduce with increasing temperature and $P_{v}$, and with decreasing μ and **P** due to a decrease in speed of sound (c) and liquid mixing [17], [18], [19]. TEC is lower for organic than aqueous solvents (∼half) and it decreases with increasing liquid height for organic medium, unlike water, due to the atomization phenomenon [20]. At the similar $P_{in}$, the batch configuration exhibits higher hydroxyl radical concentrations than the continuous flow which is a key factor for water and wastewater treatment by advanced oxidation methods [21]. An increase in transducer operation time (on/off period) using low-frequency US horn also results in higher production of free radicals, whereas the TEC remains almost constant [14].

Since sonication enhances mass transfer and reaction rates, especially in two-phase liquid, the challenge for large-scale production by industrial sectors implies the design of sonoreactor with a continuous flow. Techno-economic evaluation of USW application for biodiesel production in continuous flow was compared with conventional mechanical stirring, in which the 20.8 and 80 % reduction was observed in total investment and amount of waste produced [22]. Moreover, the high purity biodiesel was produced employing a US-assisted continuous tank reactor. Optimum transesterification conditions depended to USW amplitude, temperature, oil/methanol molar ratio, volumetric flow rate Q (space velocity ϑ) [23]. US-assisted emulsification was also shown to be a more efficient in continuous flow compared to the batch configuration [24].

Therefore, a deep understanding of what portion of $P_{in}$ is dissipated to heat ($P_{cal}$) is necessary for process efficiency purposes in batch and continuous flow. The hypothesis of complete conversion into heat is not universal in certain sonochemical processes in which some parts of supplied energy is used to perform a chemical phenomenon. To the best of the authors’ knowledge, there is no global approach to construct an engineering model to examine how acoustic energy transfer is affected by thermo-physical properties of the medium. The choice of water, sunflower oil, and their homogeneous mixtures as liquid medium allows the prediction of $P_{in}$ and TEC for any liquid with a relatively wide range of properties. Both batch and continuous flow configurations using a horn-type sonoreactor were performed to compare the empirical results with numerical simulation.

## 2 Materials and methods

Sunflower oil (Vispo brand, Iran) as an organic phase and deionized water (Tooska Shimi, Iran) as an aqueous phase were mixed at different volume fraction and then homogenized using a laboratory homogenizer (D-130, Wiggens co., Germany) to prepare a homogenous liquid mixture. All properties of the mixture were determined at the desired temperature from individual single phase. The relationships between the main properties of the liquids including ρ, μ, σ, $c_{P}$, and k in terms of temperature are given in Table S1 (supplementary material).

The schematic and real pictures of the experimental apparatus are illustrated in Fig. S1. The adiabatic sonoreactor (1 L in volume) was made of a cylindrical glass (Prisma Pyrex, Iran) with an inner diameter and height of 78 and 210 mm, respectively. It was insulated with two layers of polyethylene foam (thickness of 10 mm) and covered with aluminum foil. The container was covered with a polyethylene foam cap with a central a hole (diameter 20 mm) for the passage of the horn. A horn-type sonicator consisting of a transducer (model UP400S, 400 Watt, 24 kHz, Hielscher, Germany) and a titanium horn (H40 type) with a diameter of 40 mm, a maximum immersion depth of 70 mm, a maximum amplitude of 12 μm and a sound intensity of 12 W/cm^2^ was used to perform all experiments. An inlet and outlet flow tube (diameter of 5 mm) was installed at the bottom and top of the cylindrical glass, respectively, where the liquid flow rate was controlled by a peristaltic pump (VP200 Farayand Pardis Sina-Iran). With closing the inlet and outlet valves the continuous switches to batch-mode. There is no need to stir the liquid mixture because of streams generated by USWs. The liquid temperature in batch-mode sonoreactor was monitored using a digital K-type thermometer (UT320A, UNI-T-China) installed 10 mm next to the horn tip so as not to interfere with the USW propagation. A high precision wattmeter (EMS-EU 2.0 energy monitoring socket, Energy, England) was used to measure $P_{in}$.

USW propagation led to increase the liquid temperature since no chemical and biological reactions were occurred within the reactor in this study. TEC was determined from acoustic power ($P_{acou}$) and calorimetric power ($P_{cal}$) according to the following equation:

(1) $$TEC\;\left(\%\right)=\frac{P_{cal}}{P_{acou}}\times 100$$

The $P_{acou}$ (W) was determined from $P_{in}$ (W) which itself was measured by a power-meter, and $P_{cal}$ (W) was calculated from the following equations [14]:

(2) $$P_{cal}=\left\{\begin{array}{c} \frac{m\int_{0}^{t}C_{p}dT}{\int_{0}^{t}dt}\;batch\;configuration\\ \frac{\dot{m}\int_{0}^{t}C_{p}\left({T-T}_{in}\right)dt}{\int_{0}^{t}dt}+\frac{m\int_{0}^{t}C_{p}dT}{\int_{0}^{t}dt}\;continuous\;flow \end{array}\right)$$

where m (kg), t (s), and T (K) are the liquid mass within sonoreactor, the radiation time, and the time-dependent liquid temperature in batch configuration, respectively. $T_{in}$ (K) and $\dot{m}$ (kg/s) are the inlet liquid temperature and mass flow rate in continuous flow, respectively.

### 2.1 Assumptions

- The K and c were assumed constant within the experimental range of temperature and were taken as 4.57 × 10^-10^ m.s^2^/kg and 1480 m/s for water and 4.60 × 10^-10^ m.s^2^/kg and 1453 m/s for oil, respectively [25].

- The $P_{acou}$ was assumed to be 97 % of $P_{in}$ according to the supplier data)Hielscher, Germany(.

- All liquid properties are affected by temperature and since it is very hard to independently change a single property under a constant value for the rest (without addition of additive), the liquid properties was not considered as independent variables. Therefore, $P_{n}$ and **o/w** ratio were considered as independent variables without the need to prepare a table for design of experiment.

- The diameter of microbubbles with the average value of 10 µm was considered as characteristic length for determination of a dimensionless group known as Ohnesorge number **Oh** ($\mu /\sqrt{\sigma \rho d}$) due to the lack of information on size distribution of generated microbubbles under USWs.

## 3 Governing equations

### 3.1 Acoustic pressure

A horn-type sonicator involves three modules: a piezoelectric material, a linear elastic material, and a pressure acoustic for the transducer, horn, and water, respectively. Each module was performed by its own relations, which described the desired physics. The numerical model is developed in the finite element method (FEM)-based transient acoustic pressure module of the COMSOL Multiphysis^@^ software (V5.6, Sweden). The governing partial differential equation (PDE) used by this module to solve the acoustic wave propagation is given as [19]:

(3) $$\nabla .\left(\frac{1}{\rho \;}\nabla P_{a}\right)-\frac{\omega^{2}}{{\rho c}^{2}}P_{a}=0$$

where ω (rad/s),c (m/s), ρ (kg/m^3^), and $P_{a}$ (Pa) are the angular frequency, speed of sound in liquid, liquid density, and acoustic pressure or sound pressure level (SPL), respectively.

### 3.2 Continuity, momentum and energy balance

The law of conservation of mass in fluid dynamics (continuity equation) is written as:

(4) $$\frac{\partial \rho }{\partial t}+\nabla .\left(\rho u\right)=0$$

where u (m/s) is the liquid velocity.

The momentum equation was governed by the Reynolds-averaged Navier-Stokes (RANS) equation as follow:

(5) $$\rho \left(\frac{\partial u}{\partial t}+u∙\nabla u\right)=-\nabla P+\nabla ∙\left(\left(\mu +\mu_{T}\right)\left(\nabla u+{\left(\nabla u\right)}^{T}\right)\right)+f_{v}$$

where P (Pa), $f_{v}$ (N/m^3^), μ and $\mu_{T}$ (Pa.s) are the hydrostatic pressure, the force effects per control volume, dynamic and the turbulent liquid viscosity, respectively.

The heat transfer model defined in the software for a fluid flow as follow:

(6) $$\rho c_{P}\left(\frac{\partial T}{\partial t}+u∙\nabla T\right)=\nabla .\left(\left(k+k_{T}\right)\nabla T\right)+q_{v}$$

where $c_{P}$ (J/kg.K), $q_{v}$ (W/m^3^), **k** and $k_{T}$ (W/m.K) are the specific heat capacity of the liquid at constant pressure, the volumetric rate of energy generation due to pressure changes, cavitation and viscosity loss, the liquid thermal conductivity and the increment due to turbulence, respectively.

### 3.3 Geometry, meshing and boundary conditions

The 3D-computational domain of the batch- and continuous-mode horn-type sonoreactor was simulated with COMSOL Multiphysis^@^ software (V5.6, Sweden). A tetrahedral mesh was generated with a finer mesh size close to the US transducer probe. To ensure that the results are independent of the mesh size, a grid independent study has been carried out. The result of the mesh independence for batch mode after 360 s irradiation under $P_{n}$: 400 W with water as the media is displayed in Fig. S2. Due to the lack of significant changes in calculated temperature (308.27 K) after a convergence time of 18 h, the optimal number of the grid was chosen as 683195. The mesh quality was reported by the software as 0.7153, which was more than the minimum acceptable level for network validity, i.e., 0.65. A small discrepancy in mesh quantity and convergence time with continuous mode was exhibited.

Several modules of acoustic pressure *(acpr)*, solid mechanics *(solid),* electrostatics *(es),* heat transfer in fluid *(ht),* turbulent flow, k-ε *(spf),* and phase field *(pf)* were used to predict the wave propagation, liquid temperature, and velocity. Three solvers were also selected sequentially to perform calculations, which included frequency domain for the acoustic model, stationary for the sound current created in the fluid, and finally a time dependent for the liquid temperature. The properties of both materials including water and oil were extracted from software’s materials library. Y titanium beta-21S was considered as the US probe material, and lead zirconate titanate 9PZT-5H as the piezoelectric. The acoustic-structure boundary condition was applied to couple the pressure acoustics model to the horn solid structure that includes both the FEM and the boundary elements method (BEM) based acoustics interface. The coupling included the liquid load on the structure and the structural acceleration as experienced by the liquid. The condition on exterior boundaries was as follows [26]:

(7) $$\hat{n}.\left(\frac{1}{\rho }\left(\nabla P_{a}\right)\right)=a_{n}$$

where $\hat{n}$ and $a_{n}\left(m/s^{2}\right).$ are the unit normal vector and the normal acceleration of the liquid, respectively.

Water-glass boundary condition was set to the reactor walls which assumed to be insulated, and heat transfer between liquid and walls and between liquid and the horn was assumed to be zero. In addition, no-slip boundary conditions were used to the inner side and bottom walls of the sonoreactor. The non-isothermal multiphysics coupling was also selected to couple the heat transfer and turbulent liquid flow model. Displacements at the water-wall interface were set to zero supposing a large acoustic impedance of wall material for USW incident. Sound pressure was set to the threshold of human hearing ($2\times 10^{-6}$ Pa) for surfaces in contact with air. The liquid, horn, and transducer domains were assigned to water, piezoelectric (PZT-5H), and stainless-steel (AISI 4340), respectively.

## 4 Results and discussion

**Fig. 2:**
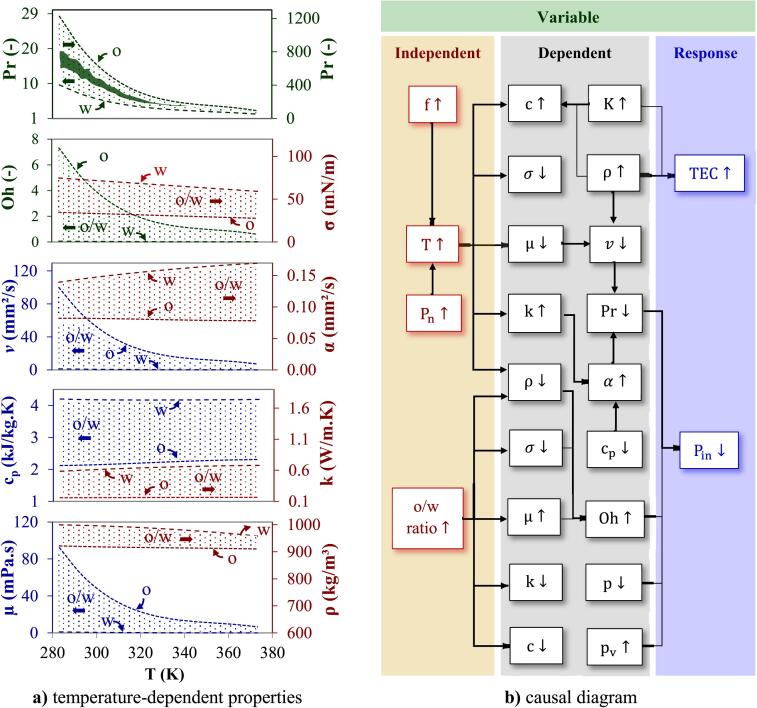
Thermo-physical properties of sunflower oil, water and their mixture **a)** temperature-dependent properties **b)** causal diagram.

The properties of the homogeneous binary mixture (shaded area in Fig. 2a) can be determined considering the changes in the individual properties of oil and water (dash lines) with temperature. The effect of temperature on μ was particularly striking due to a substantial reduction in internal friction. Compared to water, oil with higher μ exhibited a more pronounced decrease in μ with temperature. Like μ, the ρ of mixture also decreases for the temperature range of ∼ 100 K with a slighter power trend of ∼ 7 and 1 % for water and oil, respectively. However, the decrease in kinematic viscosity (v) of mixture with increasing temperature aligns with the common trend for Newtonian liquids. The difference in v for liquids with various o/w ratios is significantly greater at low temperatures (1.3 to 100.3 mm^2^/s) than at higher temperatures (0.29 to 7.4 mm^2^/s). The rise in temperature from 293 to 373 K exhibits an ascending trend in the mixture’s k and results in slight increase in thermal diffusivity (α) of both liquids and their mixture considering no significant changes in the mixture’s $c_{P}$. The Prandtl number (Pr) is a key dimensionless group in studying the thermal–hydraulic behavior of fluids and is defined as the ratio of v to α. For the investigated temperature range of ∼ 100 K, the Pr of the oil and water decreases in an exponential manner to approximately 94.41 (92 % reduction) and 1.69 (82 % reduction), respectively. The variations in Pr observed in liquids with different **o/w** ratios in the lower temperature ranges are notably influenced by the distinct behaviors exhibited by μ and $c_{P}$. Sunflower oil exhibits lower σ (∼42 mN/m) compared to water (∼78 mN/m), due to the absence of hydrogen bonding and the presence of non-polar molecules, which reduces adhesive forces. The decrease of cohesion energy determines the decline of the σ with increasing temperature, and leads to more intense bubble dynamics, such as a shorter bubble collapse time, a higher microjets speed, and a higher bubble instability [27]. Another dimensionless group, known as Ohnesorge number **Oh**, is used to describe the relative importance of viscose forces to internal and surface tension forces in fluid dynamics and to provide insights into the shape and behavior of liquid drops, bubbles, or menisci in various systems. Under the constant temperature, an increase in **o/w** ratio leads to an increase in μ and a decrease in ρ and σ and finally an increase in **Oh**. For investigated range of temperature and **o/w** ratio, the corresponding v and α were in the range 2.14 to 100.29 mm^2^/s and 0.08 to 0.17 mm^2^/s, and Pr and Oh were in the range 4.7 to 805 and 0.03 to 3.5, respectively.

The interaction among parameters affecting **P_in_** and TEC is illustrated in Fig. 2b as a causal diagram, in which the **o/w** ratio and temperature affects liquid properties such as $P_{v}$, K, ρ, μ, σ, $c_{P}$, k, v, and α. The temperature itself is affected by US frequency, which was not investigated in this study. Increasing frequency of a cup-horn transducer from low (20 kHz) to medium (500 kHz) leads to the smaller cavitation formation, higher sonochemical efficiency, and lower TEC [28]. The direct impact of **o/w** ratio (0–1), and $P_{n}$ (80–400 W) on temperature (293–365 K) and properties of liquid mixture such as ρ (914–998 kg/m^3^), μ (0.547–63.5 mPa.s), σ (28.3–72.4 mN/m), $c_{p}$ (2.13–4.18 kJ/kg.K), k (0.161–0.646 W/m.K), and their indirect impact on $P_{in}$ and TEC is the subject to be investigated in this study (Fig. 2b).

### 4.1 Wave propagation

**Fig. 3:**
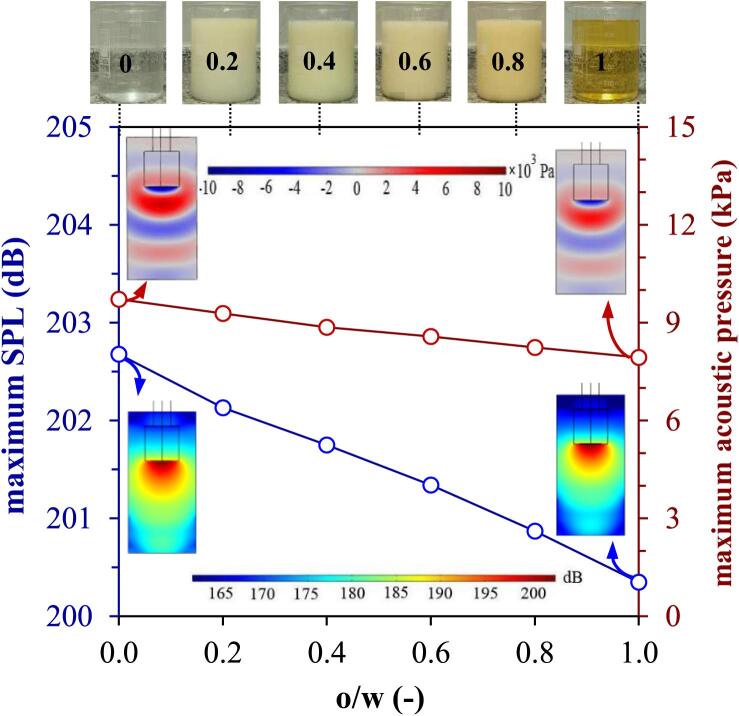
Wave propagation in homogenized mixture of various **o/w** ratios for batch configuration ($P_{n}$: 400 W).

Understanding the interactions between wave propagation and sonoreactor geometry is essential for reactor design. The sound pressure level (SPL) is an indicator of sound wave intensity in decibel (db), which is determined based on a known reference value (usually $2\times 10^{-6}$ Pa). Fig. 3 reveals the predicted maximum SPL and maximum $P_{a}$ in an **o/w** homogeneous mixture at 293.15 K and $P_{n}$: 400 W. It is obvious that by moving away from probe tip, $P_{a}$ attenuates due to the absorption of USW in liquid. The curves exhibited a decrease of ∼ 2 db and 2 kPa when changing o/w ratio from zero to one, respectively, which attributed to the lower C in oil (∼1431 m/s) compared to water (∼1498 m/s) as a denser medium [29], [30]. Therefore, to attain the same US power level, more $P_{in}$ is need for organic than aqueous solvents. In denser liquids, i.e., lower **o/w** ratio, sound waves might encounter lower resistance and lose less energy. The contours showed $P_{a}$ fluctuations around the horn tip in which the maximum SPL and acoustic pressure with different **o/w** ratios decreased symmetrically from the horn tip toward the bottom. However, the SPL changes were more pronounced compared to the changes in acoustic pressure. Transfer and distribution of US energy could create fluctuations in acoustic pressure, causing variations in its behavior and characteristics. Cavitation bubbles disperse and attenuate USWs due to the acoustic impedance of liquid ($z_{a}:\rho .c$), resulting in either partial or complete scattering of the USWs [22]. Under a constant $P_{n}$**,** more energy is required with decreasing acoustic impedance of liquid. The acoustic impedance of water increases with a maximum value at 60 °C and decreases only slightly at higher temperatures [17]. Despite more $P_{in}$**,** for liquids with higher **o/w** ratio due to its lower reflection (attributed to slightly lower acoustic impedance), the distribution of US energy within the different liquid media might differ, affecting the $P_{a}$ within each liquid. Moreover, the absorption of sound waves by the liquid can lead to lower $P_{a}$ near the bottom and corners of the sonoreactor [31]. This absorption could be higher in oil due to its different acoustic properties compared to water. The geometry at the bottom corners of sonoreactor, creates areas where sound waves might reflect and interfere with each other. This interference could lead to regions of destructive interference, causing the cancellation of sound waves and resulting in lower or even negative pressure zones [29]. An enhancement in “c” could also have an increasing effect on the emission and distribution of sound waves [32]. The progressive attenuation of USW amplitude and pressure with increasing distance from the incident wave source is because of the absorption caused by the viscous effects. Type of liquid in association with different absorption mechanisms affect USW’s attenuation which includes viscous or frictional losses, thermal conduction between the regions of dilatation and compression, thermal agitation/relaxation and energy exchange between the wave motion and the internal motion (translation, vibration, and rotation) due to the atoms collisions (Brownian motion). The reflection can occur at the interfaces of the transducers and the liquid with different acoustic impedances and affect the efficiency. Reducing the distance between horn tip and sonoreactor’s bottom resulted in a higher maximum sound pressure [33]. Therefore, a conical design of sonoreactor's bottom can be preferred to uniform distribution of sound waves, maximize cavitational volume, and minimize interference effects [31]. Recent studies also showed that a multi-stepped US horn with USW emitting from the horn neck and cone-shaped tip generated multiple zones of extortionate $P_{a}$ and a higher TEC (31.3 %) was achieved compared to a conventional cylindrical one [31], [34]. It is worth noting that no significant change was exhibited for the continuous flow compared to batch configuration (Figure not shown).

**Fig. 4:**
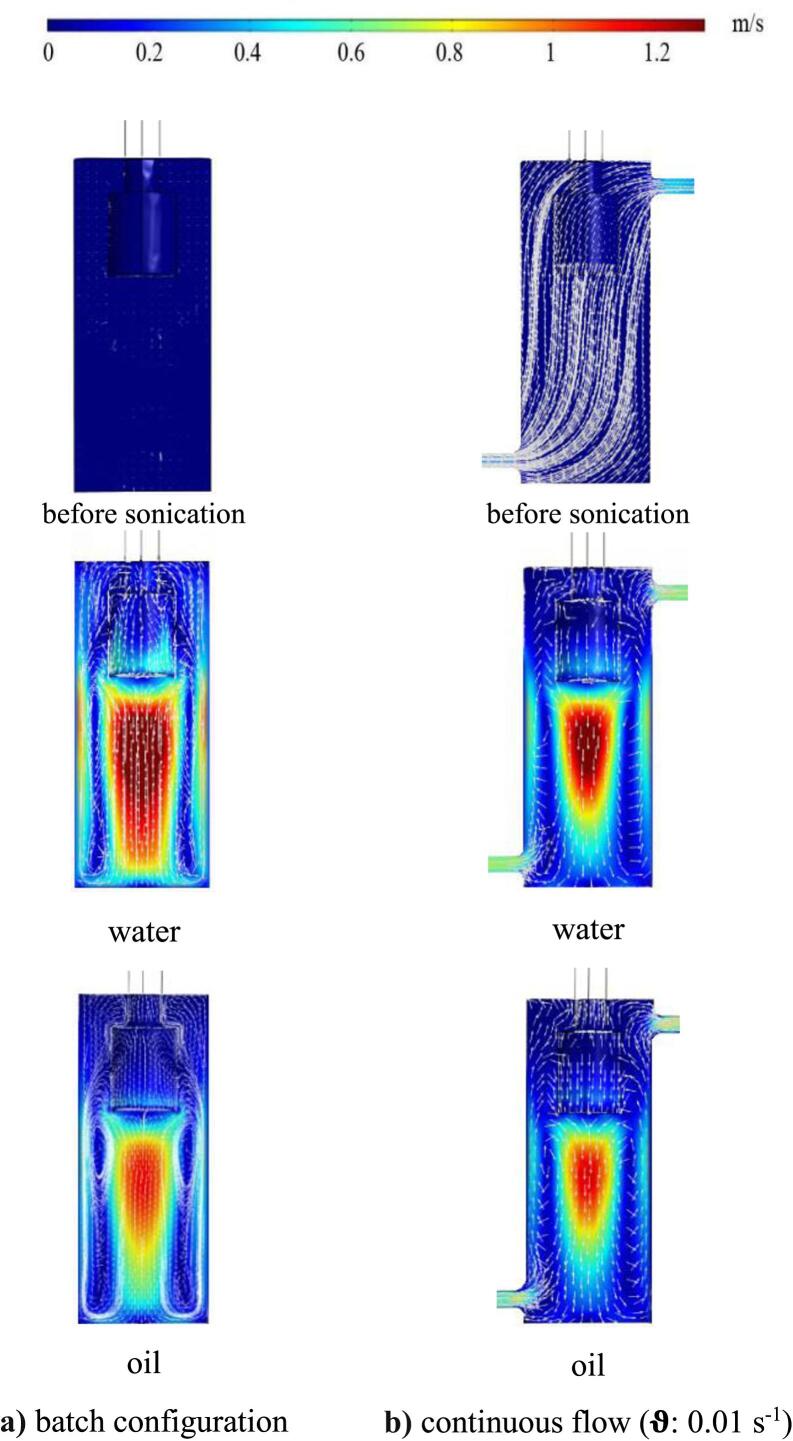
Sound velocity contour and liquid velocity vector field before and after sonication ($P_{n}$: 400 W) **a)** batch configuration **b)** continuous flow (ϑ: 0.01 s^−1^).

Sound velocity contour along with liquid velocity vector field for water and oil are illustrated in Fig. 4 for batch and continuous flow under $P_{n}$: 400 W. The liquid velocity before US irradiation was almost similar for both handled liquids (i.e., oil and water) in continuous flow with respect to the constant ϑ (0.01 s^−1^), and there was no liquid velocity vector for the batch configuration at the similar conditions. The highest and lowest sound velocity was exhibited in water and oil, respectively, for both batch and continuous flow. Therefore, a decrease in the ρ and an increase in μ, i.e., higher **o/w** ratio, caused a reduction in “c” and wave propagation due to an increase in shear stress between liquid layers. The horn tip exhibited a sound velocity pattern resembling an axial jet, revealing maximum velocity and extensive mixing underneath, contrasting with the upper liquid surface where the velocity was minimal. At the bottom of sonoreactor and near the walls, the changes in the sound velocity were very small, causing the flow to return from the side of the walls to the top against the axial jet of the liquid. The liquid flow that accelerated under the central acceleration of the probe were directed to the bottom of the sonoreactor and moved to the side under the influence of the vortex force. This return flow collided with the input flow and resulted in further mixing at the bottom of the sonoreactor. The USW caused mixing in the medium, therefore liquid velocity vectors were somewhat affected by the “c” and the directions of the vectors differed for different **o/w** ratios.

Increasing the inlet liquid flow rate in continuous flow configuration is expected to significantly influence “c” behavior and liquid flow patterns. This change is anticipated to intensify hydrodynamic effects, potentially altering sound velocity distribution within the medium. Consequently, shifts in liquid flow dynamics may intricately influence liquid velocity vectors, impacting the interaction between sound velocity patterns and liquid velocity vectors.

### 4.2 Effect of nominal power and liquid properties

**Fig. 5:**
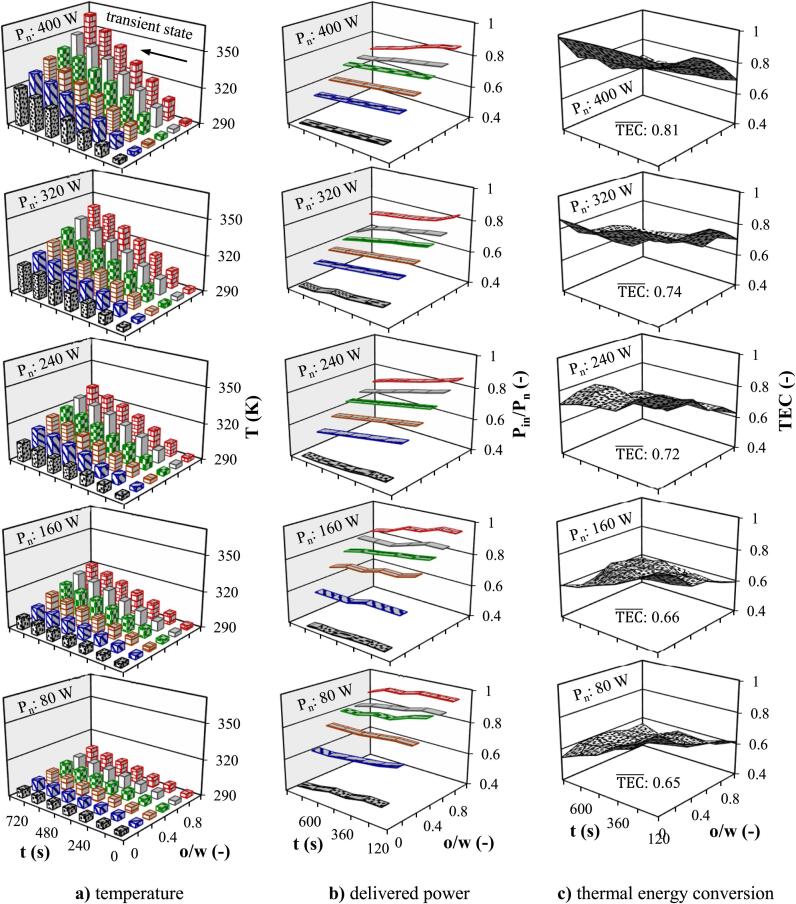
Time dependent effect of **o/w** ratio and $P_{n}$ on temperature and ultrasonic performance in batch configuration ($T_{o}$**_:_** 293.15 K) **a)** temperature **b)** delivered power **c)** thermal energy conversion.

**Fig. 6:**
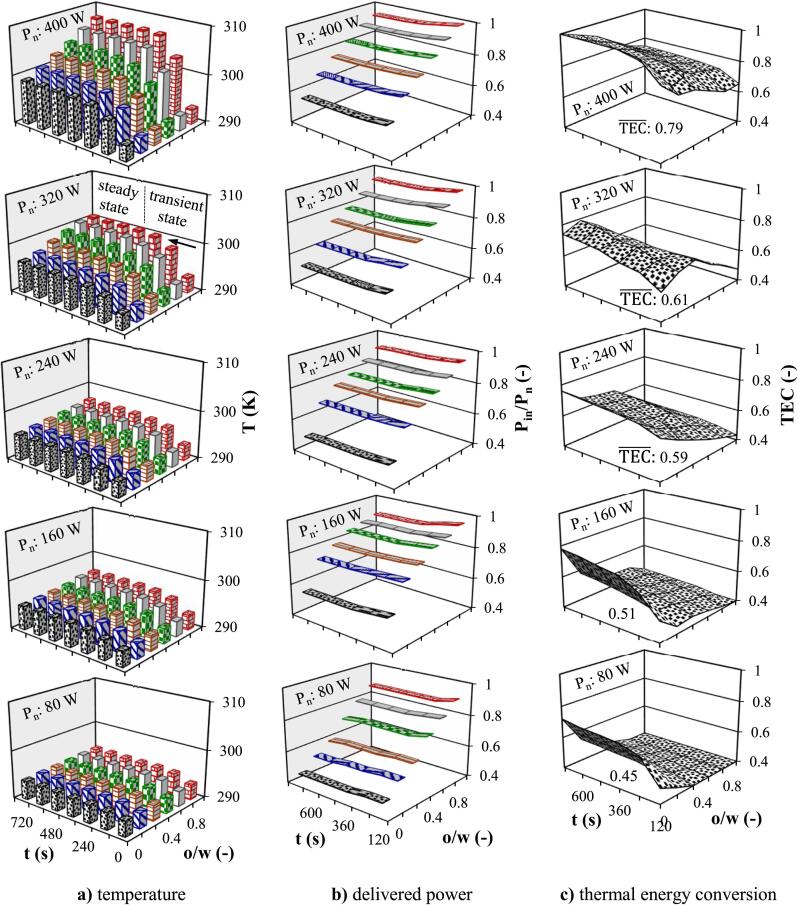
Time dependent effect of **o/w** ratio and $P_{n}$ on temperature and ultrasonic performance in continuous flow ($T_{in}$: 293.15 K, ϑ: 0.01 s^−1^) **a)** temperature **b)** delivered power **c)** thermal energy conversion.

Sonication time and power are two main factors affecting the performance of process. The effect of **o/w** ratio and US irradiation time on temperature, $P_{in}$, and TEC under varying values of $P_{n}$ from 80 to 400 W (20 to 100 % maximum amplitude) is depicted in Fig. 5, Fig. 6. Batch-mode experiments for a period of 720 s led to an increase in water and oil temperatures to a maximum value of 326 and 363 K, respectively (Fig. 5a). The volumetric heat generation due to US irradiation has been proposed to be a consequence of three phenomena, i.e., US cavitation, boundary friction generated by US vibration, and acoustic wave (mechanical energy) dissipation [35]. Among these, US cavitation has been recognized as the main cause of prominent temperature rise. A decrease in $P_{v}$ makes the cavitation bubbles smaller and thus reduces their collapse intensity. Cavitation intensity (number of cavitation bubbles per unit volume) reduces and the bubbles collapse is much more violent with increasing **ρ**, **μ**, and σ, which imply higher $P_{in}$ requirement and then facilitating the absorption of sound waves; however, μ, and σ have less influence than ρ [36]. Changes in liquid properties with increasing **o/w** ratio tend to higher **μ** and lower $P_{v}$, **ρ**, $c_{P}$, and $z_{a}$. Under the constant $P_{n}$ (e.g., 400 W), the $P_{in}$ requirement increased from 190 to 340 W with increasing **o/w** ratio from zero to one, which was attributed to the lower $z_{a}$ (more efficient energy transfer) of oil (1.3 × 10^6^ Pa.s/m) compared to water (1.5 × 10^6^ Pa.s/m) [37]. When an acoustic pulse penetrates from a piezoelectric crystal towards the liquid, depending on the $z_{a}$, part of its energy is transferred to the liquid and another part returns to the crystal as an echo. The alterations in liquid properties, created by temperature rise, enhance the cavitation intensity by reducing the resistance to bubble formation, facilitating more efficient bubble collapse, and contributing to increase sound pressure. This interaction of properties ultimately contributes to the further increase in temperature within the system, creating a feedback loop that reinforces cavitation intensity and subsequent temperature elevation. Higher $P_{n}$ can amplify acoustic effects such as cavitation intensity, collapse pressures, local temperatures [38] and also generate bigger bubbles and induce higher shear forces, contributing to increase frictional heating in the liquid [39]. A 3D computational fluid dynamics (CFD) simulation showed that the microbubbles volume augmented by ∼ 4.95 % with each 100 W increase in US power amplitude [40]. Under constant $P_{n}$, an increase in the **o/w** ratio resulted in an increase in $P_{in}$ due to an increase in μ; however, as the temperature increased over time, it slightly decreased due to a reduction in μ with temperature (Fig. 5b). The similar trend was observed for other $P_{n}$, where the cavitation bubble collapse, as the main US heating factor, will be more violent at higher $P_{n}$. On the other hand, high $P_{n}$ not only increases the acoustic energy but also decreases the threshold of cavitation. Cavitation bubbles in front of the US probe act as a barrier to the transfer of acoustic energy, reducing the power transmission to the liquid bulk [41]. Under constant **P_n_**, an increase in temperature led to a simultaneous decrease in both **P_in_** and **P_cal_**, however, the rate of decrease for **P_cal_** was greater than **P_in_**, resulting an overall decrease in TEC (Fig. 5c). The changes of TEC over time reflected the intricate interplay between temperature rise and $P_{in}$. Despite the decrease in $P_{in}$ over time for the homogenous o/w mixture in the batch configuration (Fig. 5b), the TEC remained higher for lower **o/w** ratios, indicating grater conversion of acoustic energy to heat. A slight declining TEC over time indicated a potential limit or saturation point in the US energy conversion to thermal energy. The increase in the **o/w** ratio associated with lower acoustic impedance significantly reduce TEC (∼30 %), despite the higher $P_{cal}$ of oil than water. Increasing the $P_{n}$ from 80 to 400 W in batch configuration increased the average value of TEC from 69 to 99 % for water and from 56 to 66 % for oil, respectively. Wave propagation and TEC can be affected directly by temperature changes and indirectly affected by changes in liquid properties caused by temperature changes. The reduction in **ρ** with temperature leads to enhance buoyancy due to more significant upward movement of the heated liquid, increases altered patterns of the convection currents, and also increase maximum cavitation bubble radius and raises collapse pressure [37]. Cavitation intensity decreases with increasing μ which implies a higher $P_{in}$; however, bubble collapse is more violent [17]. A significant amount of $P_{in}$ was spent to overcome liquid shear stress and liquid mixing in a highly viscous liquid, and only a part of this kinetic energy was converted into heat as viscous heating, which ultimately reduces the TEC. The oscillation amplitude of the bubbles as well as their kinetic energy decreases with increasing σ (decreasing **Oh**) and more $P_{in}$ is required for bubbles oscillation.

Limited studies have been conducted on continuous sonoreactor, particularly on production of cavitation bubbles, thermal effect, and energy conversion. Enhancing cavitation activity is important for continuous sonoreactors design due to the limited number of cavitation nuclei in the liquid [42]. The effect of $P_{n}$ and **o/w** ratio on temperature evolution was investigated in continuous flow and the results are demonstrated in bar graph of Fig. 6a for $T_{in}$: 293.15 K and ϑ: 0.01 s^−1^. The temperature experienced a rapid increase within the initial periods of US radiation (transient state) till ∼ 360 s, followed by stabilization at an equilibrium point. The turbulence liquid created by the acoustic waves causes the hydrodynamics in the vessel to approach that of a continuous stirred tank reactor, where the temperature inside the reactor is almost the same as the outlet temperature ($T_{out}$). Uniform temperature throughout the liquid medium, which was confirmed by the simulation results, indicated an efficient mixing phenomenon during US exposure. Therefore, the $T_{out}$ reliably reflected the overall temperature within the sonoreactor (T). Under similar sonication conditions, the lower liquid temperature in continuous flow than in the batch configuration created a more favorable energy transfer environment, permitting a higher $P_{in}$. Furthermore, reaching a stable temperature in a relatively short period ( 360 s) in continuous flow resulted in a consistent $P_{in}$ over time. In continuous flow, more liquid volume associated with lower temperature was irradiated over time compared to batch configuration, which contributed to the reduction of TEC. The establishment of continuous flow significantly influences the absorption and conversion of $P_{acou}$ into $P_{cal}$. The liquid turbulence was more affected by liquid flow rate, thereby causing the uniform distribution of US energy throughout the entire liquid and resulted in superior efficiency as it was shown for emulsification process [24]. In continuous flow, the influence of $P_{n}$ on TEC mirrored the same trend observed in batch configuration. Meanwhile increasing $P_{n}$ from 80 to 400 W for water in continuous flow increased the average value of TEC from 68 to 99 %, respectively, while these values for oil were from 46 to 72 % at the same conditions (Fig. 6c). Under constant $P_{n}$ in both batch and continuous flow, $P_{in}$ and temperature are higher for a homogenous mixture of higher **o/w** ratio, whereas the **TEC** is lower.

Continuous flow facilitates scalability for industrial applications and might be more practical than batch configuration as it was shown in previous publications [23], [24]. The observed temperature uniformity can also aid in better process control, especially in applications where maintaining a constant temperature throughout the process is critical for the desired outcome. The thorough mixing prevents localized hotspots or temperature gradients within the liquid, allowing for consistent energy distribution.

**Fig. 7:**
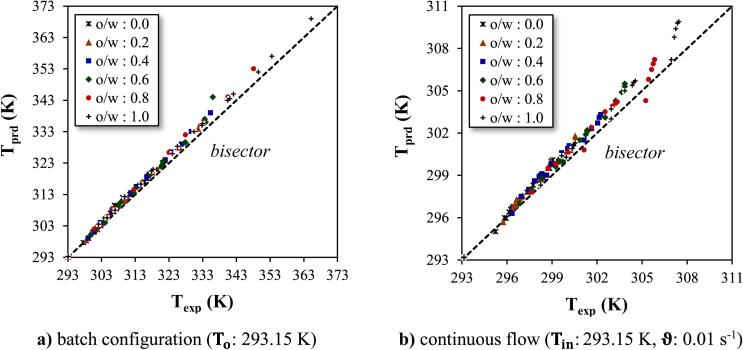
Model validation by comparison between model results and experimental data. **a)** batch configuration ($T_{o}$: 293.15 K) **b)** continuous flow ($T_{in}$: 293.15 K, ϑ: 0.01 s^−1^).

An acceptable agreement between experimental (exp.) and predicted (pred.) data obtained by the software was exhibited for temperature in batch and continuous flow (Fig. 7). However, the accuracy of the results was significantly superior in the batch compared to the continuous flow. This discrepancy indicates that the intricate processes governing US energy interaction, heat transfer, or fluid dynamics might manifest differently or be influenced by mode-specific factors, thereby impacting the accuracy of the simulation results differently in each operational mode.

### 4.3 Effect of space velocity (continuous flow)

**Fig. 8:**
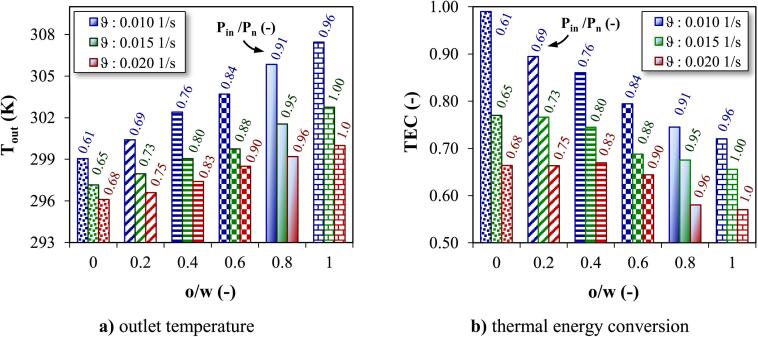
Effect of ϑ in continuous flow experiments ($T_{in}$: 293.15 K, $P_{n}$: 400 W). **a)** outlet temperature **b)** thermal energy conversion.

The effect of liquid flow rate (Q: 10 to 20 cm^3^/s) equivalent to space velocity (ϑ: 0.01 to0.02 s^−1^) on the $T_{out}$ and TEC for different **o/w** ratios under constant $P_{n}$: 400 W and continuous flow is presented in Fig. 8. An increase in ϑ led to a significant decrease in $T_{out}$ due to the shortening exposure time of liquid to US irradiation. Increasing **o/w** ratio although increased $T_{out}$ and delivered more acoustic energy to the liquid, a less part of this energy converted to heat. The decrease in TEC with **o/w** ratio was grater at lower ϑ. The ϑ can determine the intensity of the flow field in the sonoreactor, the large bubbles can split into small bubbles under the actions of the oscillating flow field, and may prompt the hydrodynamic cavitation process which does not rely on external sources like US waves [43]. A higher ϑ can create more turbulent conditions and pressure differences that might affect the stability, size, and distribution of cavitation bubbles. The bubble behavior under hydrodynamic flow conditions is transient and resembles the behavior of a cavity under acoustic cavitation. With liquid turbulence, the oscillatory stable cavitation (under stable conditions without turbulence) transforms into transient cavitation (with turbulence), similar to acoustic cavitation. Acoustic cavitating conditions as generated in ultrasonic equipments can be more simply generated in hydrodynamic flow situations by manipulating turbulence levels [44]. The highest bubble radius and bubble life are two main behaviors of generated bubbles along the liquid flow (hydrodynamic cavitation). The longer the bubble life, the more liquid is affected by the cavitation effect [44]. The induced mixing disrupts the equilibrium of the bubbles, causing some to be carried along with the flow and exit the system before undergoing complete collapse. Lower $T_{out}$ at conditions of higher ϑ also permitted a more favorable energy transfer environment, enabling a higher magnitude of $P_{in}$. Therefore, the decrease in TEC for homogenous **o/w** mixture was reduced with increasing ϑ; meanwhile, the lowest and highest TEC was related to oil (57 % for ϑ: 0.02 s^−1^) and water (99 % for ϑ: 0.01 s^−1^), respectively. It is worth to note that the temperature ($T_{out}$) is different for each experimental run (each bar) in Fig. 8.

### 4.4 General relation

**Fig. 9:**
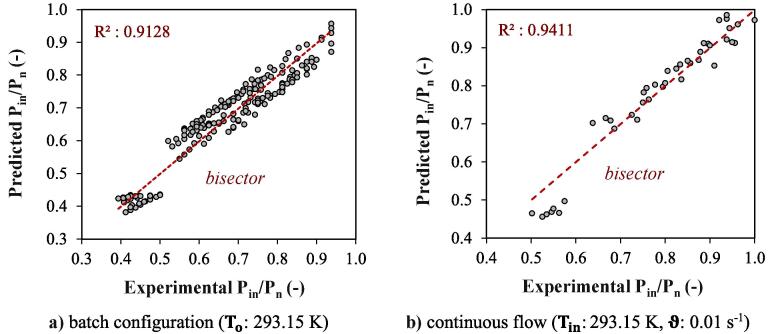
Correlation of proposed relation for input power **a)** batch configuration ($T_{o}$: 293.15 K) **b)** continuous flow ($T_{in}$: 293.15 K, ϑ: 0.01 s^−1^).

Through the comprehensive analysis of gathered experimental data in batch and continuous flow, an empirical relationship for $P_{in}$ was derived according to dimensionless groups of variables. The ϑ, temperature, **Pr**, and **Oh** numbers were used to evaluate the contribution of hydrodynamics and liquid properties on $P_{in}$. This relationship can not only serve as a model for future experiments within the reported parameters range, but can also guide experimental designs and provide a valuable tool for efficient experimental planning.

(8) $$\frac{P_{in}}{P_{n}}\left(-\right)=c_{1}{\left(1+\vartheta \right)}^{c_{2}}T^{c_{3}}{\;Pr}^{c_{4}}{\;Oh}^{c_{5}}$$

where the constants were determined experimentally as $c_{1}:128.778$, $c_{2}:7.163$, $c_{3}:-0.974$, $c_{4}:0.072$ , $c_{5}:0.089$ for both configurations. A correlation factor of $R^{2}:0.9128$ (Fig. 9a) was obtained for the batch configuration where ϑ is assumed to be zero, and a correlation factor of $R^{2}:0.9411$ (Fig. 9b) was obtained for continuous flow. The results showed that under constant temperature and **P_n_**, increasing **Pr** and **Oh** increased **P_in_** for both operating modes, while the trend was less significant for effect of **Pr** than **Oh**. Under constant **Pr** and **Oh**, increasing temperature resulted in a significant decrease in **P_in_** with a power factor closed to “-1”. Moreover, under similar **P_n_**, temperature, and liquid properties, **P_in_** was slightly higher in continuous flow compared to batch configuration. It is worth to note that changing temperature alone for a particular liquid or changing the type of liquid simultaneously changes **Pr** and **Oh** in a complex manner. For aqueous solvents with Oh < 1, surface tension forces dominate over viscous forces. Surface tension becomes the dominant factor in shaping and controlling the behavior of the liquid interface, which is often the case in small scale systems, such as microfluidic devices or capillary flows, where surface tension plays a critical role. For organic solvents with Oh > 1 surface tension forces are relatively weak compared to viscous forces. This means that the fluid's shape and behavior are primarily determined by viscosity, and surface tension effects can be neglected. For the liquid with Oh ≈ 1, both surface tension and viscous forces are significant, and their effects are comparable. This often occurs in situations where the surface tension and viscosity are both relevant in determining the behavior of the fluid interface.

**Fig. 10:**
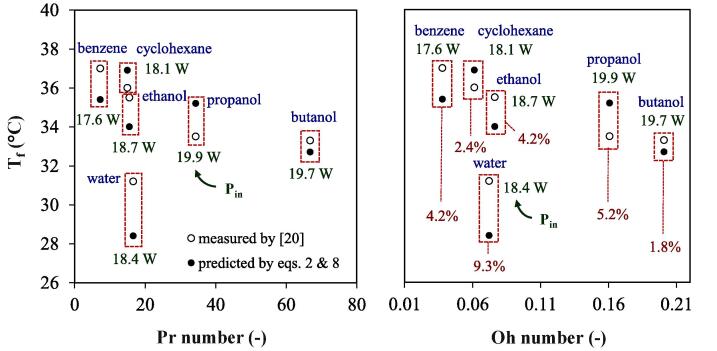
Verification of proposed correlation with the literature data [20] in a batch configuration ($T_{o}$: 25 °C, $P_{n}$: 30 W, t: 120 s).

The results obtained by Toma et al., 2011 [20] on energy conversion in a sonoreactor were used to verify the accuracy of the proposed relation. In their study, they irradiated different organic solvents (100 mL in volume) at an initial temperature of $T_{o}$: 25 °C under **P_n_**: 30 W and f: 20 kHz for a period of 120 s. Fig. 10 displays the liquid final temperature ($T_{f}$) in the batch configuration obtained experimentally and predicted using equation #8 (for **P_n_**) and then equation #2 for $T_{f}$. The highest deviation of 9.3 % was obtained for selected solvents indicating an acceptable consistence of the suggested correlation.

Attempts to derive a similar correlation for **TEC** prediction didn’t lead to a unique result due to low correlation coefficient less than 0.8. To obtain a reliable result with reasonable fidelity on **TEC** prediction, more experiments are necessary along with reducing assumptions made in this research. The fit revealed a low correlation coefficient (not shown) indicating that apart from the density, the rest of liquid properties (including **Pr** and **Oh)** had no significant effect on **TEC** for both operating modes. Cavitation field intensity decreases with increasing ρ, μ, and σ; however, the influence of liquid properties is less significant [45]. Although liquid properties influence the $P_{in}$ and TEC, it is difficult to explain these findings only based on thermo-physical properties and hydrodynamics alone.

The results of this study can be used to predict $P_{in}$ for a desired process using different liquids and to optimize acoustic cavitation for successful sonoreactor design and control.

## 5 Conclusions

There are some technical difficulties in design of an acoustic system, especially for continuous liquid flow, which prevents large-scale application. Quantitative knowledge on the parameters affecting the energy balance of sonoreactors for designing with high acoustic efficiency and low energy consumption is more important on an industrial scale. This study aimed to evaluate the conceptual relations among input power ($P_{in}$), thermal energy conversion (TEC), temperature, liquid properties using a 24 kHz horn-type sonicator. Batch and continuous flow experiments were applied for single- and homogeneous two-phase mixtures of sunflower oil and water in which no chemical effects consuming US energy occurred. Incorporating experimental and numerical approaches through COMSOL Multiphysics software, allowed for a comprehensive examination of the intricate interplay between USW propagation and energy dissipation. The direct effect of temperature on wave propagation and indirect effect on liquid properties (cavitation phenomenon) were meticulously investigated on $P_{in}$ and TEC. Under the constant nominal power ($P_{n}$: 80 to 400 W) for both batch and continuous flow, the higher **o/w** ratios led to higher temperature, $P_{in}$, and lower TEC due to changes in liquid properties, and cavitation bubble dynamics. Under similar irradiation conditions (temperature and $P_{n}$), the TEC in continuous flow were lower than that in batch configuration due to liquid hydrodynamics and disruption of the cavitation behavior. An empirical correlation for prediction of $P_{in}$ versus ϑ, temperature, Prandtl and Ohnesorge numbers was suggested. Under similar temperature and liquid properties, **P_in_** was higher in continuous flow compared to batch configuration. Higher ϑ in continuous flow led to a lower inlet/outlet temperature difference and higher $P_{in}$ as a subsequence. However, the higher ϑ resulted in a notable reduction in TEC indicating the cost-effectiveness of continuous process for commercial applications.

## Ethics approval

Not applicable.

## 7 Consent to participate

Not applicable.

## 8 Consent for publication

Not applicable.

## 9 Authors’ contributions

All authors [Jamshid Behin and Hessamodin Shahabazi] contributed to the study conception and design. Material preparation, data collection and analysis were performed by [Jamshid Behin]. The first draft of the manuscript was written by [Jamshid Behin] and all authors commented on previous versions of the manuscript. All authors read and approved the final manuscript.

## Funding

No funding was received to assist with the preparation of this manuscript.

## CRediT authorship contribution statement

**Jamshid Behin:** Writing – review & editing, Writing – original draft, Conceptualization, Data curation, Formal analysis, Supervision. **Hessamodin Shahabazi:** Software.

## Declaration of competing interest

The authors declare that they have no known competing financial interests or personal relationships that could have appeared to influence the work reported in this paper.
