# Supplementary material for: Prandtl and Ohnesorge numbers dependent of ultrasonic horn energy in Newtonian liquid under batch and continuous flow
Source: Ultrason Sonochem. 2024 Apr 3;105:106869. doi: 10.1016/j.ultsonch.2024.106869 (PMC11004692; doi:10.1016/j.ultsonch.2024.106869)
Supplement: Supplementary data 1 [file mmc1.docx]

**Prandtl and Ohnesorge numbers dependent of ultrasonic horn energy in Newtonian liquid under batch and continuous flow**

**Jamshid Behin^a,b^^[[1]](#footnote-1)^*, Hessamodin Shahabazi^a^**

^a^Faculty of Petroleum and Chemical Engineering, Razi University, Kermanshah, Iran

^b^Artificial Intelligence Division, Advanced Chemical Engineering Research Center, Razi University, Kermanshah, Iran

**Table S1.** Thermo-physical properties of single phase and homogeneous mixture of oil and water [1, 2].

| **property** | **unit** | **relationship** | **mixing rule*** |
| --- | --- | --- | --- |
| dynamic viscosity | mPa.s | $\log\mu_{w}=-26.908+{3591.145}/T+0.069T-6.456\times{10}^{-5}T^{2}$ | $\log\mu=\sum_{i:1}^{n} x_{i}{\log\mu}_{i}$ [3] |
|  |  | $\log\mu_{o}=-42.763+{6181.607}/T+0.108T-9.552\times{10}^{-5}T^{2}$ |  |
| density | $kg/m^{3}$ | $\rho_{w}=303.126\times{0.274}^{-\left( 1-T/{647.13} \right)^{0.134}}$ | $\rho=\sum_{i:1}^{n} x_{i}\rho_{i}$ [4] |
|  |  | $\rho_{o}=235.931\times{0.249}^{-\left( 1-T/{1184.45} \right)^{0.079}}$ |  |
| specific heat capacity | kJ/kg.K | $c_{\mathrm{pw}}=40.35-0.421T+18.401\times{10}^{-4}T^{2}-0.358\times{10}^{-5}T^{3}+0.262\times{10}^{-8}T^{4}$ | $c_{P}=\sum_{i:1}^{n} x_{i}{\left( \frac{\rho_{i}}{\rho_{w}} \right)c}_{\mathrm{Pi}}$ [5] |
|  |  | $c_{\mathrm{po}}=26.82-0.277T+11.300\times{10}^{-4}T^{2}-0.198\times{10}^{-5}T^{3}+0.126\times{10}^{-8}T^{4}$ |  |
| thermal conductivity | W/m.K | $\log k_{w}=-0.096-1.173\left( 1-T/{3678.45} \right)^{27}$ | $k=\sum_{i:1}^{n} x_{i}k_{i}$ [6] |
|  |  | $\log k_{o}=-0.772-0.152\left( 1-T/{4237.89} \right)^{27}$ |  |
| surface tension | mN/m | $\sigma_{w}=134.15\left( 1-T_{r} \right)^{1.6146-2.035T_{r}+1.5598T_{r}^{2}} , T_{r}=T/{647.13}$ | $\sigma=\sum_{i:1}^{n} x_{i}\sigma_{i}$ [7] |
|  |  | $\sigma_{o}$: polynomial fitting with experimental data |  |
| * n: number of phases, x_i_: volume fraction of phase i | | | |

| 40^mm^  TCI  0 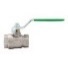 20^mm^  liquid out  210^mm^  **5**  **7**  **6**  VP200 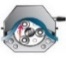 78^mm^ 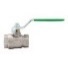 liquid in  **24 kHz**  ***UP400S*** 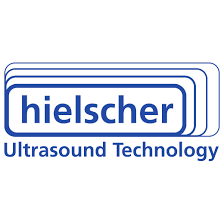 kWh  EMS 2.0  **2**  **1**  **4**  **3**  **UT-320A**  ℃ | 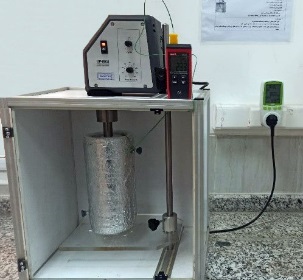 *batch* |
| --- | --- |
|  | 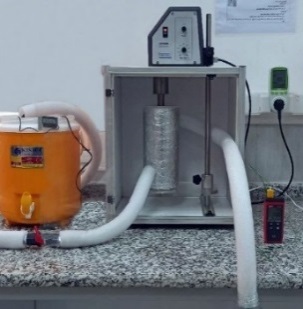 *continuous* |

**Fig. S1.** Schematic and real picture of experimental setup

(1: power-meter, 2: US transducer, 3: thermometer, 4: horn, 5: feed tank, 6: peristaltic pump, 7: sonoreactor).

**Fig. S2.** Mesh independence verification with computational geometry at selected state (mesh number: 683195, mesh size range: 1.25×10^-5^ to 1.32×10^-4^ m, convergence time: 18 h).

| **o/w**  **ratio (-)** | **time (s)** | | | | | | |  | **o/w**  **ratio (-)** | **time (s)** | | | | | | |
| --- | --- | --- | --- | --- | --- | --- | --- | --- | --- | --- | --- | --- | --- | --- | --- | --- |
|  | **0** | **60** | **120** | **180** | **240** | **300** | **360** |  |  | **0** | **60** | **120** | **180** | **240** | **300** | **360** |
| **0.0** | 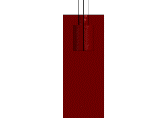 | 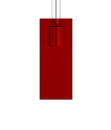 | 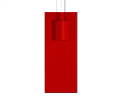 | 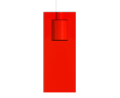 | 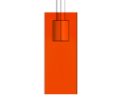 | 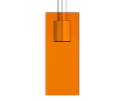 | 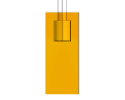 |  | **0.0** | 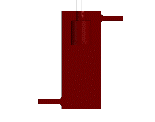 | 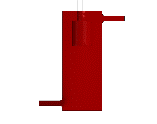 | 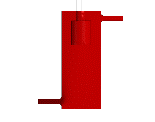 | 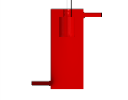 | 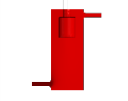 | 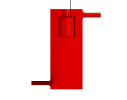 | 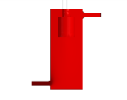 |
| **0.4** | 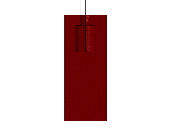 | 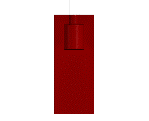 | 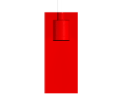 | 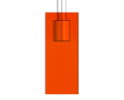 | 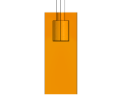 | 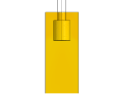 | 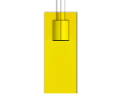 |  | **0.4** | 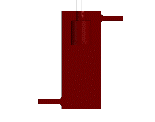 | 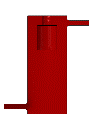 | 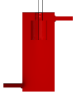 | 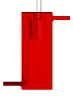 | 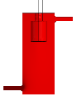 | 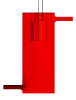 | 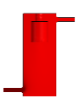 |
| **0.8** | 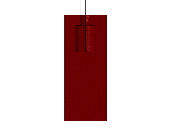 | 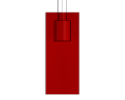 | 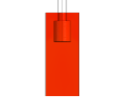 | 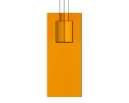 | 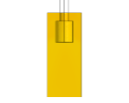 | 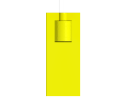 | 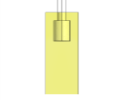 |  | **0.8** | 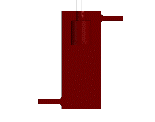 | 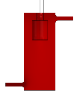 | 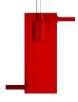 | 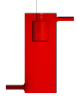 | 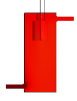 | 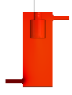 | 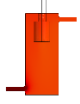 |
| **1.0** | 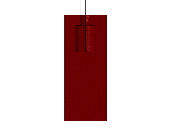 | 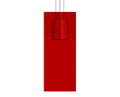 | 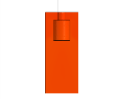 | 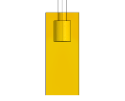 | 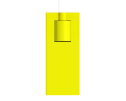 | 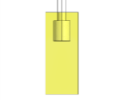 | 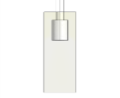 |  | **1.0** | 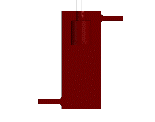 | 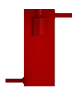 | 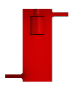 | 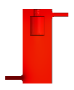 | 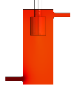 | 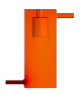 | 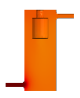 |
|  | 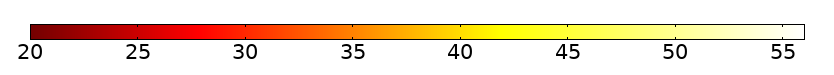°C | | | | | | |  |  | 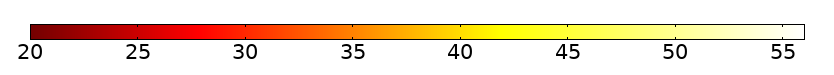°C | | | | | | |
| **a)** batch configuration (**T_o_**: 293.15 K) | | | | | | | |  | **b)** continuous flow (**T_in_**: 293.15 K, $\boldsymbol{\vartheta}$**:** 0.01 s^-1^) | | | | | | | |
| **Fig. S3.** Predicted temperature contour inside the sonoreactor. | | | | | | | | | | | | | | | | |

**References**

[1] E.E.G. Rojas, J.S.R. Coimbra, J. Telis-Romero, Thermophysical properties of cotton, canola, sunflower and soybean oils as a function of temperature, Int. J. Food Prop. 16 (2013) 1620-1629. https://doi.org/10.1080/10942912.2011.604889.

[2] [J.P. Holman, Heat transfer, Ninth edition, McGraw-Hill series in mechanical engineering, 2002.](https://sv.20file.org/up1/412_0.pdf)

[3] L. Qunfang, H. Yu-Chun, Correlation of viscosity of binary liquid mixtures, Fluid Phase Equilib. 154 (1999) 153-163. https://doi.org/10.1016/s0378-3812(98)00415-4.

[4] S. Temkin, Sound speeds in suspensions in thermodynamic equilibrium, Physic of Fluids A: Fluid Dynamics, 4 (1992) 2399-2409. https://doi.org/10.1063/1.858481.

[5] R.W. Serth, T. Lestina, Process heat transfer: Principles, applications and rules of thumb, Academic press, 2014.

[6] P.S. Puri, J.M. deMan, Thermal conductivity of liquid-liquid emulsions, Can. Inst. Food Technol. J. 10 (1977) 49-52. <https://doi.org/10.1016/s0315-5463(77)73436-4>.

# [7] J.G.J. Eberhart, The surface tension of binary liquid mixtures, Phys. Chem. 70 (1996) 1183-1186. <https://doi.org/10.1021/j100876a035>.

1. [↑](#footnote-ref-1)
